# Facemasks Readily Available in Common Stores used by Nepalese, How much Protection is Enough Protection against COVID-19?

**DOI:** 10.31729/jnma.6024

**Published:** 2021-02-28

**Authors:** Neela Sunuwar, Anu Radha Twayana, Santosh Katuwal, Swotantra Gautam

**Affiliations:** 1 B.P. Koirala Institute of Health Sciences, Dharan, Nepal; 2 Kathmandu University School of Medical Sciences, Dhulikhel, Nepal

**Keywords:** *COVID-19*, *facemask*, *protection*

## Abstract

During the COVID-19 pandemic, the Centers for Disease Control and Prevention and World Health Organization strongly recommend that people wear face masks to cover their mouths and noses while they are out and about in any other public area. There are a lot of masks available on the market, and people get a lot of mixed messages about what is safe. This article explores what kind of facemasks are readily available in Nepali General stores and what are the things to keep in mind before buying a mask. We will also discuss how many times a face mask can be used, proper ways to store them, correct ways to use facemasks, and the rationale behind its use.

## INTRODUCTION

The sole purpose of mask use is the intention of source control (preventing an infected person from transmitting the virus to others) and/or protection (preventing a healthy wearer from the infection).^1^ While relaxing the lockdown in Nepal, the Ministry of Home Affairs (MoHA) has appealed to wear facemasks as per Centers for Disease Control and Prevention (CDC) guidelines and follow safety measures to slow down the spread of coronavirus, including a physical distance of two meters.^2^

Box 1. **Current CDC guideline for wearing a mask.^3^**

Wear a mask over your nose and mouth

- Masks help prevent you from getting or spreading the virus.
- You could spread COVID-19 to others even if you do not feel sick.
- Everyone should wear a mask in public settings and when around people who don't live in your household, especially when other social distancing measures are difficult to maintain.
- Masks should not be placed on young children under age 2, anyone who has trouble breathing, or is unconscious, incapacitated, or otherwise unable to remove the mask without assistance.
- Do NOT use a mask meant for a healthcare worker. Currently, surgical masks and N95 respirators are critical supplies that should be reserved for healthcare workers and other first responders.
- Continue to keep about 6 feet between yourself and others. The mask is not a substitute for social distancing.

## FACEMASKS EASILY AVAILABLE IN GENERAL STORES OF NEPAL

There is quite a large pool of options of facemasks available one can choose from.

Basic cloth face mask, surgical face mask, N95 respi rator, Filtering facepiece respirator, P100 respirator/gas mask, Self-contained breathing apparatus, Full face respirator, Full-length face shield, KN95 respirator are few to mention.^4^ Among them surgical face mask one time use disposable and 3ply, KN95 respirators with and without valves, and basic cloth face mask are widely available in Nepal.^5^ Table 1 shows a comparison of important parameters and their performance on different masks.^6^ A study conducted by Neupane et al. on locally available facemasks in Nepal showed that the filtering efficiency of Cloth Maks ranged from 63% to 84%. The filtering efficiency of the surgical mask was found to be 94%. They concluded the major difference in efficiency is larger pore size in cloth facemask and any factors that contribute to the enlargement of pore size like washing, drying, and stretching.^7^ The fabric materials tested in a study conducted by Rengasamy et al. revealed marginal levels of respiratory protection for 20-1000 nm aerosols (droplet nuclei) (SARS-CoV-2 virion size approximately 50-200 nm in diameter).^8^ The use of improvised fabric materials may be of some value compared to no protection because fabric materials would not suffer from limited supplies, unlike respirators and surgical masks for emergency protection.

**Table 1:** Comparison of different kinds of masks.^6^

Mask Type	Affordability	Efficiency	Sealing and fitting	Light weight	Comfortable	Respiratory filter	Reusable	Mobility	Durability
Cloth mask	High	Low	Low	High	High	No	Yes	High	Mod
Surgical face mask	High	Mod	Low	High	High	No	No	High	low
Full-length face shield	High	Mod Low for indirect aerosols	Low	High	Moderate	No	Yes	High	Mod
N95	High-Mod	High	Mod	High	High	Yes	No not suitable for washing	High	Low
P100 respirator	Low	High	High	Moderate	Moderate	Yes	Yes	Low	High
Self-contained breathing apparatus	Low	Excellent	Excellent	Low	Moderate	Yes	Yes	Low	High
Full face respirator	Low	Excellent	Excellent	Low	Low	Yes	Yes	Low	High

## HOW TO WEAR A FACEMASK FOR OPTIMUM PROTECTION

Before buying a facemask, Nepalese general stores and people must consider the availability, durability, cost, and optimum protection from its use.

According to CDC following things must be considered before buying a face mask:

- Fits snuggly but comfortably against the side of the face
- Secures with ties or ear loops
- Includes multiple layers of fabric
- Allows for breathing without restriction
- Able to be laundered and machine dried without damage or change to the shape

The criteria for various medical face masks include material performance testing for bacterial filtration efficiency, differential, pressure resistance to synthetic blood, flammability, and submicron particulate filtration efficiency.^9^ But in the case of a normal day to a day face mask, instead of focusing on whether or not a mask has a filter, one must ensure that the mask is made from multiple layers of fabric (The gold standard is having three layers of fabric, where the outer layer is water repellent (e.g., polyester), the inner layer is absorbent (e.g., cotton), and the middle layer is a blend of two fabrics. But even just one type of fabric with three layers works,^10^ fits tightly on the face, and fully covers the nose and mouth. Individuals should wash or sanitize their hands before and after touching the mask. When wearing a mask, we should avoid touching it or taking it off to speak to others.

## STORAGE

There is no proper way of storage or disposal for cloth masks or any approval agencies. Once removed, it is advised to place the mask in a zip lock bag until the next wash, which is advised after each wear. In the case of respirators, the storage conditions and shelf life are usually found on the bottom or side of the primary package. Storage conditions are depicted as a thermometer for temperature range and an umbrella for humidity. For example, “When stored in original packaging between temperatures from -4 °F (-20 °C) to +86 °F (+30 °C) and not exceeding 80% RH, the respirator may be used until the date specified on packaging located next to the “Use by Date” symbol."^11^

## HOW TO REUSE

The best strategy is to isolate it for a week in a breathable container such as a paper bag to let the mask air dry for seven days except for a cloth facemask, requiring only two days. Putting masks in the plastic may lead to the accumulation of moisture and the development of bacteria. Surgical masks should not be washed because liquid damages the filter.

**Best Coronavirus Face Mask**

For the average person who isn't working with patients in a healthcare setting, a cloth face covering is the best mask for coronavirus. According to the University of Maryland here are some of the reasons why:^12^

- They can easily be made using supplies at home or purchased.
- They can be used repeatedly with regular washing between wears.
- You are not at risk of purchasing counterfeit, ineffective masks.
- They arenot a scarce resource needed by health care workers.

Cotton checks off all those boxes. It's easy to clean and comfortable to wear while at the same time offering the most protection.^13^ Also, by using a cotton facemask, we will not be taking away from any medical professionals, rescue teams, or people with breathing issues by opting for this type of mask.

## DISADVANTAGES OF FACEMASK

According to World Health Organization (WHO) guidelines following are disadvantages of facemask:^1^

The potential disadvantages of mask use by healthy people in the general public include:

- Headache and/or breathing difficulties, depending on the type of mask used
- Development of facial skin lesions, irritant dermatitis, or worsening acne, when used frequently for long hours
- Difficulty with communicating clearly, especially for persons who are deaf or have poor hearing or use lip reading
- A false sense of security leading to potentially lower adherence to other critical preventive measures such as physical distancing and hand hygiene
- Poor compliance with mask-wearing, in particular by young children
- Waste management issues; improper mask disposal leading to increased litter in public places and environmental hazards
- Disadvantages for or difficulty wearing masks, especially for children, developmentally challenged persons, those with mental illness, persons with cognitive impairment, those with asthma or chronic respiratory or breathing problems, those who have had facial trauma or recent oral maxillofacial surgery, and those living in hot and humid environments.

## MYTHS REGARDING FACEMASK USE AMONG NEPALESE

The ongoing myth about facemask among Nepalese has quite many similarities with people of the United States and other countries.^14^ Here are few myths that are prevalent in Nepal-

**Myth: Cloth masks have limited protection.**

Fact: Cloth face masks create a barrier between the mouth and nose and surrounding people. This makes it more difficult for the droplets that spread coronavirus through coughs, sneezes and talking to reach other people. It prevents unknowingly spreading the disease to others and serves as an indirect reminder to avoid touching the face. Some studies indicate it can even protect from large droplets as well. This is why wearing a cloth mask in public places was made mandatory by the Ministry of Home Affairs of Nepal (MoHA) and the ministry has sent a circular to authorities concerned to take actions against such people in line with the provisions of the Infectious Disease Act, 2020, BS.^2,15^

**Myth: There are masks of superior quality than cloth masks.**

Fact: Every type of masks has its own merits and demerits. General public and COVID-19 patients should wear a cloth mask to conserve personal protective equipment (PPE) for medical workers.

**Myth: Wearing Masks can cause suffocation.**

Fact: Some people have suggested that carbon dioxide from exhaling gets trapped under the cloth causing discomfort and suffocation, which is false. Properly fitted masks offer adequate airflow making the accumulation of carbon dioxide impossible. However, people with breathing problems, children under age 2, and those who can't remove the mask without assistance should not wear one.

**Myth: Only the sick should wear a mask.**

Fact: According to the CDC, asymptomatic people who have a disease can unknowingly spread it, including those with underlying conditions who have a higher coronavirus risk and are more vulnerable to severe illness. They can spread the disease without taking proper precautions, including wearing a mask, washing their hands frequently, and social distancing.

**Myth: Do not wear a mask while at home**

Fact: It is true, but it's important to wear a mask to protect people who feel sick, have mild symptoms, and live with others.

**Myth: Those who recovered from the corona virus do not need a mask.**

Fact: After recovery, there is a certain amount of immunity from the virus, but it is unknown if all have immunity or how long your immunity might last. People might have re-infection and become a carrier. Everyone should wear a cloth mask when in public unless they have breathing problems, are under age two, or can't remove the mask without assistance.

**Myth: No need for a mask when outside.**

Fact: At this time, being outside is generally considered safer than being inside. When taking a stroll or participating in other outdoor activities alone, a mask isn't required. If it is difficult to maintain 6 feet apart, a mask is required.

**Myth: You can go outside freely if you wear a mask.**

Fact: Masks are just one strategy to prevent the spread of corona. Wearing a mask is highly effective and safe, but it's not a permission slip to “return to normal.” It's important to stay home when one can.

## WAY FORWARD

Face Masks are easily available in Nepali general stores. Disposable surgical facemask, KN95 facemask, and cloth facemask are most widely used, but the CDC guidelines of proper use of masks have not been followed.
